# AneRBC dataset: a benchmark dataset for computer-aided anemia diagnosis using RBC images

**DOI:** 10.1093/database/baae120

**Published:** 2024-12-25

**Authors:** Muhammad Shahzad, Syed Hamad Shirazi, Muhammad Yaqoob, Zakir Khan, Assad Rasheed, Israr Ahmed Sheikh, Asad Hayat, Huiyu Zhou

**Affiliations:** Department of Information Technology, Hazara University Mansehra, Dhodial, Mansehra, Khyber Pakhtunkhwa 21120, Pakistan; Department of Computer Science, National University of Sciences and Technology (NUST), Kach Road, near Sheikh Mohammad Bin Zayed Al Nahyan Cardiac Centre, Quetta, Balochistan (NCB) 87300, Pakistan; Department of Information Technology, Hazara University Mansehra, Dhodial, Mansehra, Khyber Pakhtunkhwa 21120, Pakistan; School of Physics, Engineering & Computer Science, University of Hertfordshire, College Lane Campus, Hatfield, Hertfordshire AL10 9AB, UK; Department of Information Technology, Hazara University Mansehra, Dhodial, Mansehra, Khyber Pakhtunkhwa 21120, Pakistan; Department of Computer Science, National University of Sciences and Technology (NUST), Kach Road, near Sheikh Mohammad Bin Zayed Al Nahyan Cardiac Centre, Quetta, Balochistan (NCB) 87300, Pakistan; Department of Information Technology, Hazara University Mansehra, Dhodial, Mansehra, Khyber Pakhtunkhwa 21120, Pakistan; Department of Pathology, Shaukat Khanum Memorial Cancer Hospital and Research Centre (SKMCH&RC), 7 A Khayaban-e-Firdousi, Block R3 Block R 3 M.A Johar Town, Lahore, Punjab 5400, Pakistan; Department of Pathology, Shaukat Khanum Memorial Cancer Hospital and Research Centre (SKMCH&RC), 7 A Khayaban-e-Firdousi, Block R3 Block R 3 M.A Johar Town, Lahore, Punjab 5400, Pakistan; School of Computing and Mathematical Sciences, University of Leicester, University Road, Leicester LE1 7RH, UK

## Abstract

Visual analysis of peripheral blood smear slides using medical image analysis is required to diagnose red blood cell (RBC) morphological deformities caused by anemia. The absence of a complete anaemic RBC dataset has hindered the training and testing of deep convolutional neural networks (CNNs) for computer-aided analysis of RBC morphology. We introduce a benchmark RBC image dataset named Anemic RBC (AneRBC) to overcome this problem. This dataset is divided into two versions: AneRBC-I and AneRBC-II. AneRBC-I contains 1000 microscopic images, including 500 healthy and 500 anaemic images with 1224 × 960 pixel resolution, along with manually generated ground truth of each image. Each image contains approximately 1550 RBC elements, including normocytes, microcytes, macrocytes, elliptocytes, and target cells, resulting in a total of approximately 1 550 000 RBC elements. The dataset also includes each image’s complete blood count and morphology reports to validate the CNN model results with clinical data. Under the supervision of a team of expert pathologists, the annotation, labeling, and ground truth for each image were generated. Due to the high resolution, each image was divided into 12 subimages with ground truth and incorporated into AneRBC-II. AneRBC-II comprises a total of 12 000 images, comprising 6000 original and 6000 anaemic RBC images. Four state-of-the-art CNN models were applied for segmentation and classification to validate the proposed dataset.

**Database URL:**  https://data.mendeley.com/datasets/hms3sjzt7f/1

## Background and summary

A precise and proactive approach reduces the worst results in medical treatment procedures. Most of the worldwide disease rate goes to the chronic stage due to the nonavailability of precise and early diagnostic approaches, especially in developing countries. Anemia is the seventh leading cause of disability in women and 12.8% of maternal deaths [1]. Meanwhile, pathology tests act as bases for treating and diagnosing disease. Approximately 70% of the decisions related to the diagnosis and treatment of disease rely on the pathology test results. In this regard, different blood count and morphology tests are considered a base for diagnosing various diseases. These diseases are related to blood morphology disorders (including anemia, leukemia, polycythemia, etc.) and the immune system (including allergy, autoimmune anemia, etc.) [2]. Blood morphology disorders include several medical conditions that affect the size, shape, structure, and overall appearance of blood cells. Anemia is the most common type of blood morphology disorder. The chief cause of anemia is the nutritional deficiency in children and infants [3]. Iron deficiency anemia is the most common type worldwide among the different kinds of anemia disease. In South Asian countries like Pakistan, Bangladesh, and India, the economic burden due to anemia is reported as $4.5 billion annually [4, 5].

**Figure 1.**
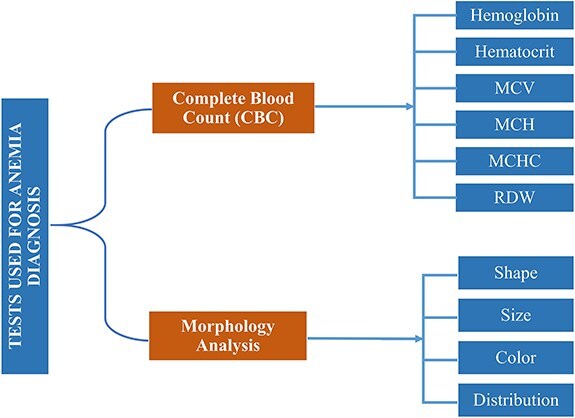
Clinical tests complete-blood-count (CBC) and morphology analysis are used for anemia diagnosis.

In reference [6], authors reported that around 600 million school-aged and preschool children are affected by anemia globally [7]. According to the World Health Organization (WHO) [8], 65.5% of preschool children in Southeast Asia are anemic. Based on mean corpuscular volume (MCV) and mean corpuscular hemoglobin concentration (MCHC) of complete blood count (CBC), anemia can morphologically be categorized into three groups, i.e. microcytic, normocytic, and macrocytic hypochromic anemia [9]. MCV measures the average volume or size of individual red blood cells (RBCs) in a blood sample. It is calculated by dividing the total volume of RBCs by their number, while MCHC measures the average concentration of hemoglobin in a given volume of RBCs. Both are key indices used in the CBC to help diagnose and classify different types of anemia and other blood disorders. Based on morphological disorder due to anemia, RBC can be classified into five subgroups, i.e. normal, microcytes, macrocytes, elliptocytes, and target RBCs [10]. Clinical tests used for anemia diagnosis [11] based on CBC and morphology are listed in Fig. 1.

Currently, both manual and automated methods are used to evaluate these tests. Manual methods include analysis under the microscope, while Sysmex XP-300, Nihon Kohden Blood Cell Counter, and DH36 3-Part Auto Hematology Analyzer are used for automated blood cell analysis [2]. Hematology Analyzers is a medical laboratory instrument used to measure and count blood cells along with other components in blood samples. It is based on the principle of light scattering and electrical impedance.

Besides the high cost and resources required for these devices, CBC counting without structural, qualitative, and morphological evaluation is another severe downside. So, after CBC through these hematology analysers, they prepare peripheral blood smear slides to evaluate structural, qualitative, and morphological disorders under the microscope. The microscopic evaluation was carried out by pathologists manually. During manual blood cell analysis, pathologists have faced several issues. Some of them are given below.

- They required outstanding expertise and rich experience for accurate microscopic blood cell analysis.
- Pathologists face challenges when identifying or predicting abnormal cell structures at the pixel level using a simple microscope [11].
- Pathologists also faced difficulties identifying the percentage change of infected cells to the normal using a simple/compound microscope.
- Approximately 5–10 min are required for the examination of one slide.
- Overlapped structures hide the density of blood elements in unit volume and morphological features (shape and size) of abnormal RBCs to normal.
- Pathologists cannot accurately differentiate between normal and abnormal shapes and sizes using a compound microscope, where RBCs show overlapped structures.
- The manual analysis required resources and dedicated concentration to examine the blood sample.
- Manual analysis can be affected due to the errable nature of humans, like tiredness, domestic problems, etc.
- Current blood analyzers perform CBCs but do not facilitate pathologists in examining the blood cell morphological structure at the pixel level.

These factors may lead to the incorrect diagnosis and treatment of the disease. However, penetration of bioimage analysis using a deep neural network and image processing techniques improves the structural, qualitative, and morphological analysis even on the pixel level [2]. Medical image analysis penetrates multiple domains, including quantitative microscopy, digital pathology, systems pathology, and computational pathology [12–17]. Image processing techniques rapidly expand bioimage analysis through technological advancement in imaging modalities regarding images’ morphological, structural, texture, and pixel-level analysis. Furthermore, publicly available medical image databases and platforms allow the research community to access image datasets and annotations to develop sophisticated algorithms to analyze complex medical images [18].

Over the past few decades, several techniques and datasets have been proposed for the morphological analysis of RBCs and white blood cells (WBCs). In medical image analysis, segmenting the region of interest (ROI) is the initial rudimentary step for image processing [19]. Based on the efficient segmentation of blood cell elements like WBCs, RBCs, and platelets, numerous analyses can be carried out after the segmentation, like feature extraction, classification of blood cell elements, etc. These analyses provide valuable information during morphological analysis and disease diagnostic procedures [20]. In reference [21], Nikitaev *et al*. introduced a technique for segmenting leukocytes from blood and bone marrow cells. A total of 1018 cell images were formed out of 50 samples. In references [22–24], authors proposed algorithms based on marker-controlled watershed and thresholding, morphological-based techniques for systematically segmenting leukocytes and erythrocytes. Due to their close proximity, blood cells may overlap in microscopic images of blood smears. In reference [25], the authors try to overcome the problem of overlapping cells using image processing techniques and neural networks. Cao *et al*. [26] performed a deep study on the segmentation of leukocytes in peripheral blood smears. They introduced an algorithm SWAM&IVFS (stepwise averaging method) considering RGB and HIS color space. In reference [27], the authors proposed an image-processing algorithm for classifying WBCs and nuclei based on the nuclei features. In reference [28], authors classify RBCs based on their shape. They introduced an outlier tolerant machine learning (ML) technique to determine the blood’s slipper-shaped and croissant-shaped cells. The deep neural network used in references [29–31] to segment MRI and CT scan images, while [32, 33] classified the WBCs and RBCs, respectively, to detect sickle cell anemia. Another study [3], explores the diagnosis of three types of anemia, i.e. (i) iron deficiency anemia (IDA), (ii) α-thalassemia trait, and (iii) β-thalassemia trait, by using WEKA software. The author used two parameters to evaluate the data mining technique, i.e. highest accuracy and lowest mean absolute error. They combined the J48, IBK, and Naïve Bayes algorithms with vote algorithms and got accuracy scores of 96.343 and 96.2169 to diagnose iron deficiency anemia. They analyzed blood smear images of 793 individuals, containing 184 IDA patients (71 males and 118 females), 409 thalassemia patients (α-thalassemia 206 and β-thalassemia 203), and a control group of 200 healthy individuals (100 males and 100 females). In reference [5], the authors analyzed the classification approaches, i.e. decision tree (KNN, CNN, SVM, logistic regression) and association rule mining for statistical analysis of anemia [11]. Veterans Affairs Informatics and Computing Infrastructure database were used in reference [34] to find the anemia risk to be within a 95% confidence interval of 74.95%–75.26%. Random prediction (Rp) classification algorithms were used in reference [35] to select anemia in pregnant women.

The experimental data used to develop and authenticate all the above algorithms is microscopic blood images. The availability of state-of-the-art blood image datasets is pivotal in developing deep ML models. We need diverse state-of-the-art image data, annotations, and ground truth to train and test deep ML models to diagnose and classify different diseases. To develop a curated dataset, the generation of ground truth, annotations, and labeling of images should be carried out under the supervision of a team of expert pathologists. Developing a fine-tuned dataset for medical purposes is challenging and time-consuming, but it plays a crucial role in smartening the medical diagnostic process. Unfortunately, limited blood image databases are available for the research community to evaluate blood cell morphology. These databases are ALL-IDB-I, ALL-IDB-II [36], extended ALL-ID [37], BCCD, IUMS-IDB [38], SMC-IDB [39], BS_DB3 [40], Ash bank, BBBC [41], peripheral blood cell image dataset [42], RBC counting automation [43], malarial-infected blood RGB images [44], and Raabin-WBC [2]. Most of these datasets are not publicly available. The publicly available datasets are composed of a small number of images with short information related to blood cell morphology. As an example, ALL-ID-I contains only 108 images with only 39 000 blood elements. All images have no masks to authenticate segmentation results. All datasets have no ground truth for the authentication of proposed techniques except extended ALL-IDB [37] and Raabin-WBC [2]. Other datasets also have no expert metadata, annotation, or labeling of images that may help to diagnose the disease accurately. The comparative analysis of all these datasets is given in Table 3.

**Table 3.** Comparative analysis of proposed AneRBC-I, and AneRBC-II datasets with previously developed blood cell datasets

Dataset	No. of images	Blood elements	Resolution	Ground truth	CBC reports	Morphology reports
ALL-IDB-I [36]	109	WBC & RBC	2592 **× **1944	**×**	**×**	**×**
ALL-IDB-II [36]	260	WBC	257 **× **257	**×**	**×**	**×**
SMC-IDB [39]	367	WBC	Nil	**×**	**×**	**×**
IUMS-IDB [38]	196	WBC	Nil	**×**	**×**	**×**
Malarial dataset	848	RBC	Nil	**×**	**×**	**×**
AneRBC-I Dataset	1000	RBC	1224 × 960	1000: binary 1000: RGB	**✓**	**✓**
AneRBC-II Dataset	12 000	RBC	306 × 320	12 000: binary 12 000: RGB	**✓**	**✓**

**Figure 2.**
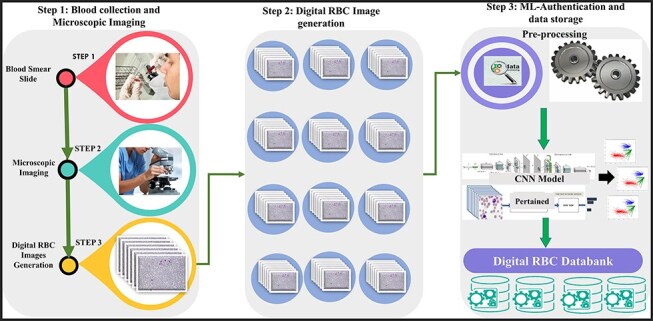
The complete schematic overview of blood collection to dataset development.

We introduced a state-of-the-art benchmark AneRBC-I and AneRBC-II datasets to overcome these challenges and automate the morphological analysis of blood cells. We target the morphological disorder of RBCs due to anemia. The Schematic overview of this study is given in Fig. 2.

The key characteristics of the AneRBC dataset are listed below:

### A large number of images

**Figure 3.**
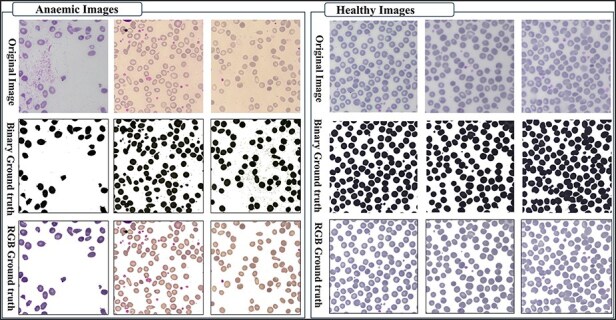
Original images along with segmented binary, and RGB ground truth of healthy and anemic samples.

The AneRBC-I dataset has 1000 original blood images, including 500 normal and 500 anemic images. These 1000 images were further processed, and 2000 manually generated 1000 binary and 1000 RGB segmented ground truths were obtained, as shown in Fig. 3. So, the total number of images included in the AneRBC-I repository are 3000 (1000 original, 1000 binary segment, and 1000 RGB segmented). The size of the image is 1224 × 960. Each image contains approximately 1550 RBC elements. So, the original 1000 images comprise approximately 1 550 000 RBC elements. These elements include normocytes, microcytes, macrocytes, elliptocytes, and target RBCs. The AneRBC-II dataset has a total of 36 000 images. Out of these, 12 000 are original images, including 6000 normal and 6000 anemic images. The remaining 24 000 included 12 000 binary segmented and 12 000 RGB segmented images.

### Ground truth for result authentication

**Figure 4.**
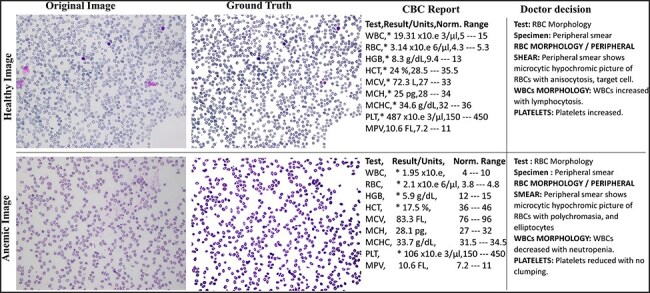
Original and RGB segmented ground truth samples of healthy and anemic images including original, ground truth, CBC report, and doctor decision in AneRBC-I dataset.

The AneRBC dataset is equipped with two different types of ground truth for each image, i.e. binary segmented ground truth and RGB segmented ground truth, as shown in Fig. 4. These ground truths were generated under the supervision of a team of expert pathologists at the pathology lab of Shaukat Khanum Memorial Cancer Hospital and Research Centre (SKMCH&RC) in Lahore, Pakistan. These ground truths will help the research community to train and authenticate more generic CNN models. Manually generated ground truth for the ROI plays a crucial role in evaluating segmentation and classification models.

### Abnormal RBC morphology

The images in both the AneRBC-I and II datasets are divided into two subgroups: healthy and anemic images. This classification will assist the research community in training deep learning models to identify morphological disorders in RBC. Furthermore, the research community can also use this dataset to classify Anemic RBC images into microcytes, macrocytes, elliptocytes, and target cells.

### CBC and morphology reports

Another distinct characteristic of the AneRBC datasets is the availability of CBC and morphology reports of each image. Based on our understanding, the AneRBC dataset represents the first instance of a blood image dataset that includes both CBC and morphology reports for each image, enabling the automated anemia diagnosis decision to be compared against clinical test results.

### Evaluation of deep neural network models

AneRBC-I dataset is equipped with 1000 original microscopic images, including 1 550 000 healthy/anemic RBC elements, and ground truth, CBC, and morphology report of each image. Furthermore, AneRBC-II comprises 12 000 original RBC images. This data repository enables the research community to evaluate the performance of deep neural networks for the precise detection of morphological disorders in blood cells.

### Diversity of lighting effects for generalized ML models

All the images were collected using a single microscope but with different lighting effects. Images with heterogeneous background lighting effects help to evaluate the performance of generalized ML models.

## Methods

The methodology adopted for the development of this dataset comprises on two phases. (i) Patient selection and peripheral blood smear collection at SKMCH&RC lab, and (ii) manual slide analysis and image capturing with heterogeneous lighting intensity. The research conducted in this study follows the ethical guidelines and has received approval from the Internal Review Board (IRB) of Shaukat Khanum Hospital, Lahore, Pakistan, and Hazara University Mansehra. The IRB carefully reviewed and endorsed the research methodology, ensuring that it fulfills ethical standards and protects the rights and well-being of the participants. All participants involved in this study provided informed consent before participating, and they were explicitly informed about the nature of the research, its objectives, and the intended use of their data. Furthermore, participants were guaranteed that their identities, like name, affiliation, age, gender, etc., would remain confidential, and steps were taken to protect their privacy throughout the study. Particularly, each participant agreed to the open publication of the data, understanding that it would contribute to advancing knowledge in the respective field.

### Patient’s selection and peripheral blood smear collection

After obtaining approval from the ethical committee of Shaukat Khanum Memorial Cancer Hospital and Research Centre (SKMCH&RC) Lahore, Pakistan, 1000 participants were selected from the pathology lab at SKMCH&RC to provide peripheral blood smears for the study. Out of which, 500 were healthy, and 500 were anemic participants. To reduce gender inequality, we have selected 50% male and 50% female from each group, i.e. healthy and anemic. The age range for both males and females was 30–50 years. The summary of the population is given in Table 1. Peripheral blood smear collection and slide preparation were done under the supervision of a team of expert pathologists from the SKMCH&RC laboratory. The volume of the blood that was taken from each patient was 3.0 ml. If sample transportation is required, 24:00 ho were given with 2°C–8°C temperature. The blood sample was preserved in a Lavender cap EDTA tube.

**Table 1.** Patient population description of both anemic and nonanemic

Category	Total patients	Male	Female	Age range
Anemic	500	250	250	30–50
Nonanemic	500	250	250	
Total	1000	500	500	

The sample was received at the laboratory, and clots were checked before further processing. After that, these samples were processed on a Sysemex xn9000 analyzer to generate CBC and morphology parameters. Slides of each sample were prepared with Wright-Giemsa stain for microscopic analysis. Olympus Dp27 8.9-megapixel CMOS sensor camera captured the RBC images with a 4M resolution. Sample images from healthy and anemic blood elements are shown in Fig. 3. The binary ground truth represents a black-and-white segmented version, where the foreground (RBC elements) and the background are distinctly marked. The RGB ground truth includes color segmented images with only foreground (i.e. RBC elements) without the background. Both the binary and RGB ground truths were generated manually using segmentation software under the supervision of expert pathologists from Shaukat Khanum Memorial Cancer Hospital and Research Centre (SKMCH&RC). The binary ground truth was created by selecting the RBC regions with a high-precision tool, while the RGB ground truth used color coding for detailed cell morphology classification.

### Manual slide analysis and image capturing with heterogeneous lighting intensity

After preparing blood smear slides, pathologists from SKMCH&RC lab examined the slides under an Olympus Dp27 8.9-megapixel CMOS microscope. The study was supervised by a team of two senior consultant pathologists and three assistant consultant pathologists. The senior consultants brought extensive experience and knowledge in the field of pathology, particularly in the interpretation of blood cell morphology and the diagnosis of hematological disorders. Their supervision ensured the quality and accuracy of the pathological assessments conducted as part of the study. Additionally, the assistant consultant pathologists, while perhaps less experienced than their senior counterparts, provided valuable support in data collection, analysis, and review. Together, the combined expertise of the pathologist team ensured rigorous and thorough examination of the blood cell images, contributing to the credibility and reliability of the study outcomes.

The pathologists examined all the slides and gave appropriate interpretations of morphological disorders in the cells. The pathologist’s interpretation was also stored in the database with morphology reports, as shown in Fig. 4. So, in this way, we have generated 1000 CBC and morphology reports of all images and pathologists’ interpretations for ML Models performance evaluation. After that, images were captured with 1224 × 960 pixels resolution and heterogeneous lighting intensity to develop a more diverse blood image dataset. All images were stored on storage media in PNG format.

### Ground truth generation

The AneRBC dataset includes two distinct types of ground truth for each image: binary segmented ground truth and RGB segmented ground truth, as shown in Fig. 4. Initially, these ground truths were generated using Photoshop. We used the magic tool for the selection of the background with a 20% tolerance. The manually generated ground truth was authenticated under the supervision of a team of expert pathologists at the pathology lab of Shaukat Khanum Memorial Cancer Hospital and Research Centre (SKMCH&RC) in Lahore, Pakistan. These annotations serve as valuable references for the research community for the training and validation of deep learning CNN models. Manually generated ground truth for the ROI plays a crucial role in evaluating segmentation and classification models. The detail of both Binary and RGB ground truth is given below.

#### Binary ground truth

Binary ground truth annotations are typically used to denote the presence or absence of specific features within an image. In the case of the AneRBC dataset, binary annotations likely indicate the boundaries and presence of RBCs against the background. These annotations are usually in black and white (binary), where white (or one) represents the background and black (or zero) represents the ROI. Binary annotations are often created using image segmentation tools that allow pathologists or trained technicians to outline the cell boundaries manually or semi-automatically. For this purpose, we have used Photoshop software with magic tool for the selection of the background with a 20% tolerance. This method ensures precise delineation of cells, which is crucial for segmentation tasks in ML.

#### RGB ground truth

RGB ground truth involves more detailed annotations that include color information. This type of annotation is used to differentiate various types of cells or cell states based on additional attributes like color and texture. In the context of anemia, different colors in the RGB annotations might represent various types of RBCs such as normocytes, microcytes, macrocytes, elliptocytes, and target cells.

## Dataset description

**Figure 5.**
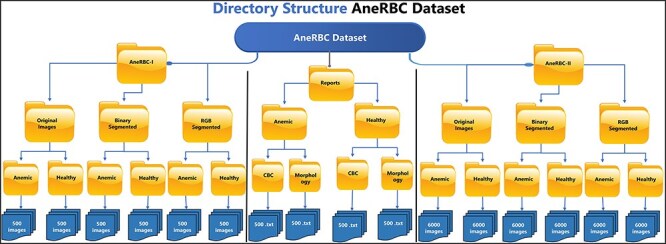
The directory structure of the AneRBC Dataset.

The AneRBC dataset is divided into two distinct versions based on image resolution and type: (i) AneRBC-i and (ii) AneRBC-ii. The director structure and the number of images in each directory of the AneRBC dataset are shown in Fig. 5.

### AneRBC-I

AneRBC-I consists of 1000 original images (500 healthy and 500 anemic images) with a resolution of 1224 × 960 pixels. These 1000 original images are further processed, and 2000 images are generated with 1000 binary and 1000 RGB ground truths, as shown in Figs 3 and 4, respectively. The CBC and morphology reports of all 1000 images with the pathologist’s interpretation are also included in the database. Resultantly, 3000 images (1000 original, 1000 binary, and 1000 RGB ground-truth) were stored in the AneRBC-I data repository along with CBC, morphology, and doctor decision report of each image.

### AneRBC-II

**Figure 6.**
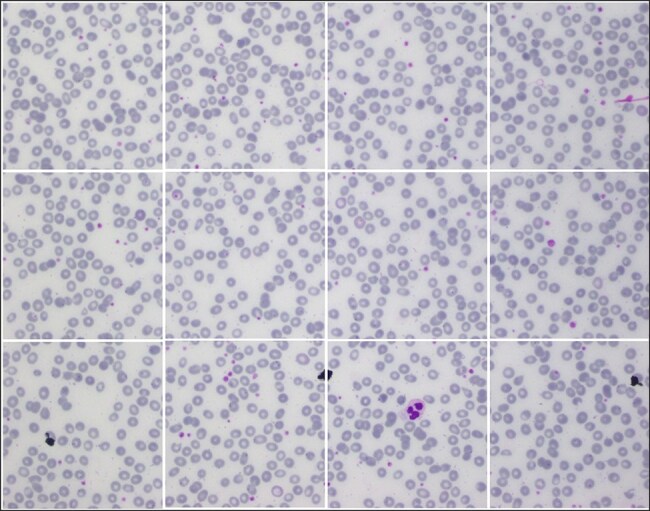
Samples images from AneRBC-II dataset, where 1224 × 960 is divided into 12 subimages with size 306 × 320.

Due to high resolution, most ML Models cannot process these images in the current size, i.e. 1224 × 960. So, each image is cropped and divided into 12 images with a resolution of 306 × 320 pixels. As a result, AneRBC-II comprises 12 000 original images (6000 healthy and 6000 anemic images) along with 12 000 binary and 12 000 RGB ground truths. Resultantly, AneRBC-II stored a total of 36 000 (12 000 original, 12 000 binary, and 12 000 RGB ground truth) images, as shown in Fig. 6. A short description of both datasets is given in Table 2.

**Table 2.** Detailed description of both versions of the AneRBC datasets

Type	Image acquisition setup	
Camera	Olympus Dp27 8.9-megapixel CMOS	
Magnification of microscope	40×	
Image format	PNG	
Color Depth	3 (RGB)	
**Dataset**	**AneRBC-I**	**AneRBC-II**
Total no. of images	3000	36 000
Original	1000	12 000
Binary ground truth	1000	12 000
RGB ground truth	1000	12 000
Resolution	1224 × 960	306 × 320
Image type	50% anemic and 50% healthy	50% anemic and 50% healthy
Ground truth	Yes	Yes
CBC reports	Yes	Yes
Morphology and doctor decision reports	Yes	Yes

### Image name representation

The AneRBC-I images are named with annotations xxx_a.png and xxx_h.png. Here xxx is a three-digit integer counter, “a” represents images with anemic RBC elements, and “h” represents healthy elements. The naming annotation for AneRBC-II is xxxx_yy_a.png and xxxx_yy_h.png. The AneRBC-II dataset is the subdivision of AneRBC-I. Each image in AneRBC-I is divided into 12 subimages. So, here “xxxx” represents the 4-digit counter for the total number of images, while “yy” represents the counter for each image’s subdivision. The naming representation of relevant manually segmented images is also the same, but the segmented images are placed in a separate directory named “segmented.”

### Blood cell element types

**Figure 7.**
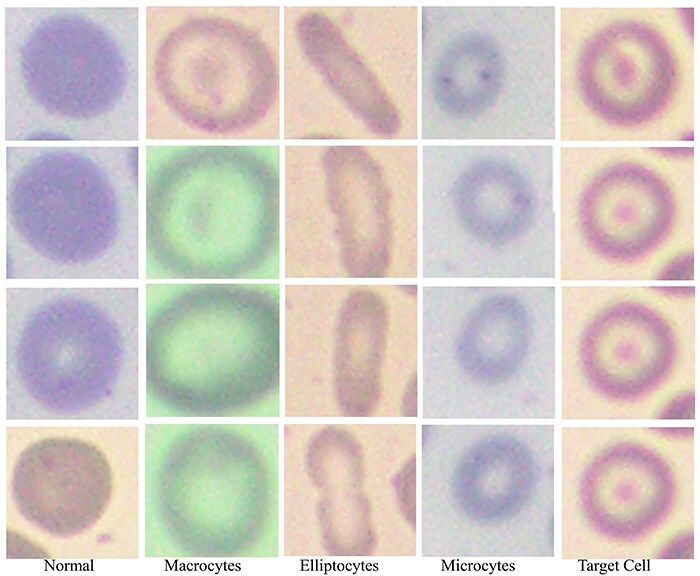
Red blood cells in the anemic images can be classified as normal, macrocytes, elliptocytes, microcytes, and target cells.

Based on RBC morphology, the AneRBC dataset is divided into healthy RBC images and anemic RBC images. The anemic RBC images can be further categorized into five classes, i.e. normal, microcytes, macrocytes, elliptocytes, and target RBCs, as shown in Fig. 7. Each image in the AneRBC-I dataset contains approximately 1550 RBC elements, including five classes. So, our dataset comprises approximately 1 550 000 RBC elements. Each image in the dataset, either original or ground truth, is provided in .PNG format in AneRBC-I and II.

### Dataset comparisons

The comparative analysis of proposed datasets with previously developed blood cell datasets like ALL-IDB-I, ALL-IDB-II [36], extended ALL-ID [37], BCCD, IUMS-IDB [38], SMC-IDB [39], BS_DB3 [40], Ash bank, BBBC [41] is given in Table 3.

## Technical validation

CNNs remain a preferred choice due to their abilities to capture spatial hierarchies in image data. We have conducted experiments to authenticate the proposed dataset by segmenting and classifying the RBC elements and then validating the results against the ground truth. To set the baseline for deep learning-based instances and semantic segmentation, we compared the segmented ROI of the RBC element with manually generated ground truth. Furthermore, three types of technical validation were carried out on the proposed dataset: clinical validation, segmentation validation, and classification validation. Alternative ML approaches such as support vector machines (SVMs) and decision trees offer several advantages in certain contexts, but they generally lack the spatial hierarchies in image data that CNNs effectively capture. Therefore, tasks involving complex image analysis like medical image segmentation, CNNs remain preferred choice due to automatically detect patterns and features directly from image pixels.

### Clinical validation

Initially, the proposed dataset was clinically verified by the consultant pathologists. The ground truth of each healthy and anaemic image was generated during this verification under the pathologist’s supervision. In parallel, each image’s morphology and decision report were also added to this dataset, which was generated using the CBC report by the consulted pathologists.

### Segmentation validation

The technical validation of the AneRBC-I and AneRBC-II datasets is critical in establishing their utility for the research community, particularly for segmentation tasks in medical imaging. The application of standard convolutional neural network (CNN) models, including UNet, LinkNet, and AttenUNet, along with a generative adversarial network (GAN), provides a robust framework for evaluating the datasets’ quality and the feasibility of their use in developing advanced segmentation algorithms We have applied four state-of-the-art CNN models to validate both datasets concerning segmentation, including GAN, UNet, LinkNet, and AttenUNet models. A detailed description of the results obtained from each model is given below.

#### UNet model performance

##### AneRBC-I

The standard UNet model demonstrated an impactful performance on the AneRBC-I dataset, with a Dice Coefficient of 0.9414 and an intersaction over union (IoU) of 0.8916, indicating a high degree of overlap between the predicted segmentation and the ground truth. The accuracy of 0.9174 is commendable; however, it is not the highest among the tested models. The model’s precision and recall are closely matched (0.9332 and 0.9448, respectively), suggesting a balanced segmentation capability. The specificity of 0.8982 indicates that UNet identifies true negatives effectively using the proposed dataset, which is crucial for medical image segmentation tasks.

##### AneRBC-II

On the AneRBC-II dataset, the UNet model’s performance improved across all metrics, with a prominent increase in the Dice Coefficient to 0.9693 and IoU to 0.9407. The accuracy also raised to 0.9585. This evaluation metric enhancement could be attributed to the diverse dataset’s characteristics to generalize the given data better. The precision and recall remained high, with values above 0.96, reinforcing the model’s reliability in segmenting the AneRBC-II dataset.

#### LinkNet model performance

##### AneRBC-I

LinkNet outperformed UNet in the AneRBC-I dataset, achieving a higher accuracy of 0.9426 and the best Dice Coefficient (0.9497) and IoU (0.9083) among the models. The precision and recall were the highest at 0.9474 and 0.9557, respectively, indicating a superior segmentation quality with the AneRBC-I dataset. The specificity of 0.9232 was also the highest, showing that LinkNet was most adept at correctly identifying negative cases.

##### AneRBC-II

In the AneRBC-II dataset, the performance of LinkNet slightly increased as compared to its results on AneRBC-I for all metrics except precision and specificity as shown in Tables 4 and 5. The accuracy increased slightly from 0.9426 to 0.9447, Dice Coefficient from 0.9497 to 0.9535, IoU from 0.9083 to 0.9129, and recall from 0.9557 to 0.9629. The model maintained a high Dice Coefficient of 0.9535 and an IoU of 0.9129, but both UNet and AttenUNet outperformed it in most metrics.

**Table 4.** The loss, accuracy, Dice Coefficient, IoU, precision, recall, and specificity for UNet, LinkNet, and AttenUNet models, demonstrating the technical effectiveness of AneRBC-I dataset for the medical image segmentation task

Model	Loss	Accuracy	Dice coefficient	IoU	Precision	Recall	Specificity
Unet	0.2503	0.9174	0.9414	0.8916	0.9332	0.9448	0.8982
LinkNet	0.2018	0.9426	0.9497	0.9083	0.9474	0.9557	0.9232
Atten_Unet	0.2719	0.9780	0.9829	0.9665	0.9833	0.9825	0.9700

**Table 5.** Loss, accuracy, Dice Coefficient, IoU, precision, recall, and specificity for each CNN model. The results validate the AneRBC-II dataset’s robustness and applicability for developing and testing advanced segmentation models in the medical image processing domain

Model	Loss	Accuracy	Dice coefficient	IoU	Precision	Recall	Specificity
Unet	0.1358	0.9585	0.9693	0.9407	0.9698	0.9689	0.9368
Linknet	0.1851	0.9447	0.9535	0.9129	0.9462	0.9629	0.9221
Atten_Unet	0.1660	0.9812	0.9743	0.9634	0.9415	0.9264	0.8924

#### AttenUNet model performance

##### AneRBC-I

AttenUNet showed better results on the AneRBC-I dataset, with the highest accuracy of 0.9780 and specificity of 0.9700 among the three models. The Dice Coefficient and IoU were outstanding at 0.9829 and 0.9665, respectively. These results suggest that the attention mechanisms integrated into AttenUNet boost the identification of relevant features for segmentation.

##### AneRBC-II

Interestingly, while still excellent, AttenUNet’s performance on the AneRBC-II dataset was not as dominant as on the AneRBC-I dataset. The accuracy remained high at 0.9812, but there was a decrease in the Dice Coefficient to 0.9743 and IoU to 0.9634. The precision and recall show a little decline, with recall dropping to 0.9264, which was the lowest among the models for this dataset.

#### Conditional Generative Adversarial Network

**Figure 8.**
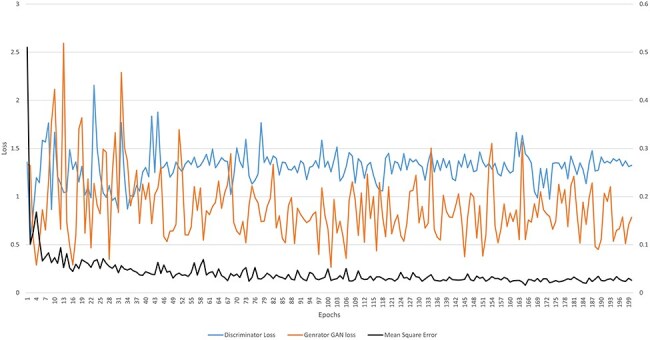
Graph of discriminator loss, generator loss, and mean square error.

**Figure 9.**
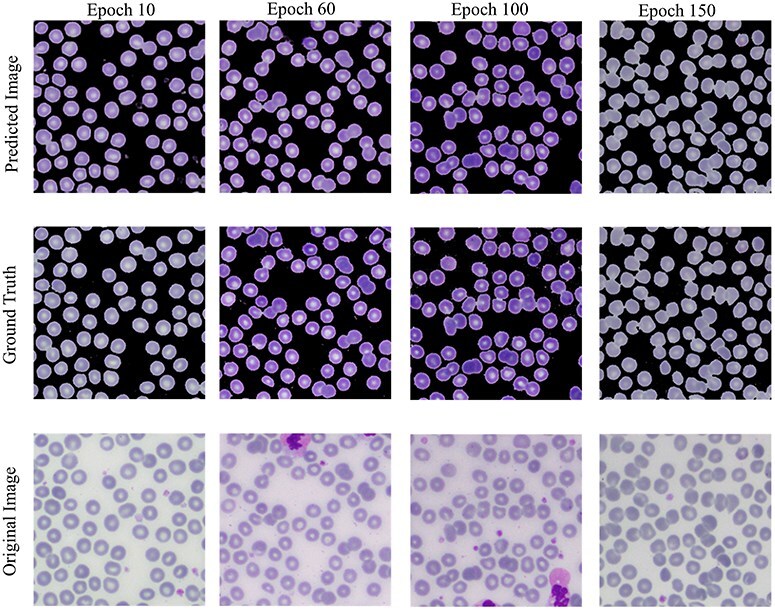
Visual results of image segmentation using the GAN network at different epochs.

The Conditional Generative Adversarial Network (cGAN) network comprises a generator and a discriminator. The generator generates a predicted image against each input image, while the discriminator discriminates between the predicted image and ground truth. Unlike traditional GANs that generate images purely from random noise inputs, the cGAN used in our research takes an actual image as input and produces a segmented image as output, aligning closely with the goals of medical image segmentation. The results related to the discriminator, generator losses, and mean square error are shown in Fig. 8. This figure shows that after 15 epochs, the predicted image does not look realistic. An epoch is one complete pass through the entire training set by the learning algorithm. The predicted image looks more natural as epochs increase from 10 to 50. As the epochs increased upto 80, we got optimized results. This phenomenon indicates that the proposed AneRBC-II dataset is technically valid for the research community. The results of the GAN network were evaluated using the different loss functions. The visual representation of segmentation is shown in Fig. 9.

The reason for GAN network is used for the segmentation task of the proposed datasets is twofold: (i). Using GANs alongside traditional UNet architectures for segmentation allows for the evaluation of the dataset’s utility; (ii) Successful segmentation with both UNet and GAN architectures acknowledges the credibility and applicability of the dataset for a broader range of image analysis tasks.

The generator loss determines how accurately the ROI is segmented in the predicted image near the ground truth. Generator loss can be is determined using the following formula.


1 $${G_L} = \,{E_x}\left[ {log\left( {D\left( x \right)} \right)} \right] + {E_z}\left[ {log\left( {1 - D\left( {G\left( z \right)} \right)} \right)} \right]$$

where *G* and *D* represent the generator and discriminator function of GAN, respectively. *G(z)* and *D(x)* are the output of generator and discriminator probability estimation, respectively. While *[log(D(x))]* is the output of discriminator on real data. The generator minimizes its loss in this function to generate a more realistic image near the ground truth. Figure 8 shows that the highest generator loss during execution was 2.155 at epoch no 23, while it reduced to 1.322 at epoch no 200.

The discriminator loss maximizes the prediction probability related to the generated image as fake. Mathematically, it is represented as:


2 $${D_L} = maximize\log D\left( x \right) + \log \left( {1 - D\left( {G\left( z \right)} \right)} \right)$$

where *D(G(z))* represents the discriminator’s output when evaluating fake data. The term *log(D(x))* shows the log of discriminator identification. Whereas the term *log(1-D(G(z)))* expresses the discriminator correctly identifying generator-produced data as fake. Figure 8 shows that the highest discriminator loss during execution was 2.592 at epoch no 13, while it reduced to 0.763 at epoch no 198.

The mean squared error (MSE) between generated image (g) and ground truth (${g_0}$) is calculated as given below.


3 $$\begin{array}{*{20}{c}}
{MSE = \frac{1}{n}\mathop \sum \limits_{i = 1}^n {{\left( {{g_0} - g} \right)}^2}}
\end{array}$$

where the term $\mathop \sum \limits_{i = 1}^n {\left( {{g_0} - g} \right)^2}$ shows the squared differences from i = *1* to *n*. MSE measured for the AneRBC dataset is shown in Fig. 8. The maximum MSE of 0.510 was calculated at the 1st epoch and reduced to 0.0158 at epoch no 165.

**Figure 10.**
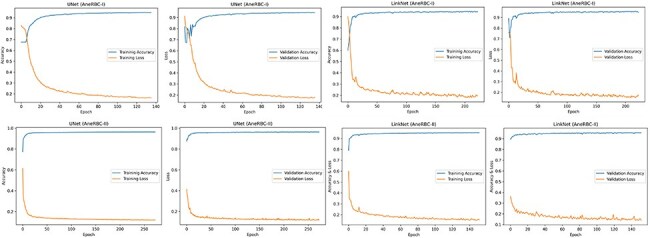
Training and validation graph for Unet and LinkNet models.

**Figure A1.**
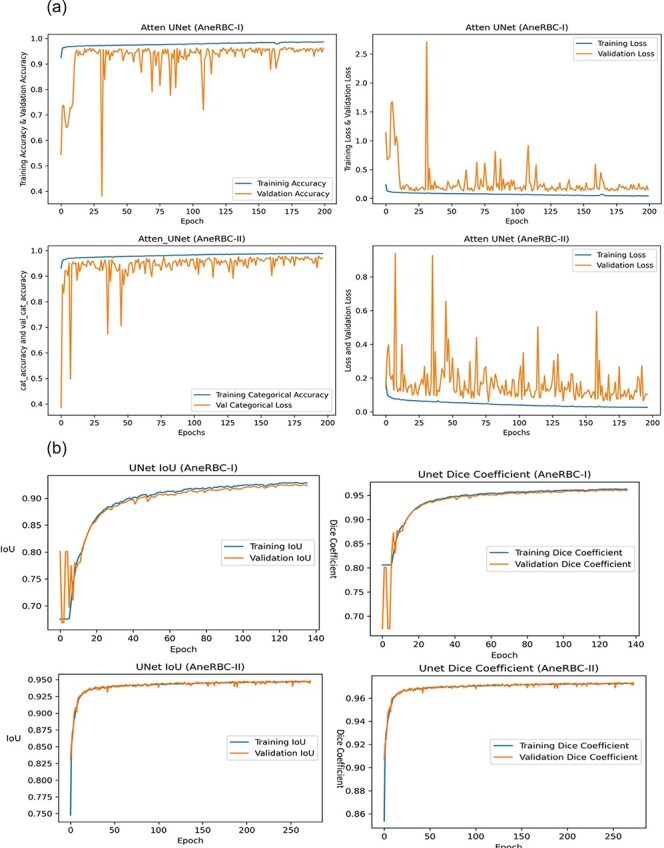

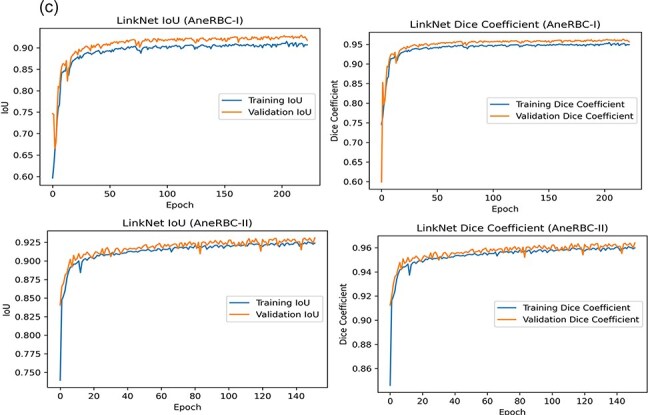
(a) Training progress of Atten_UNet on AneRBC-I and AneRBC-II. (b) IoU and Dice-Coefficient of UNet on AneRBC-I and AneRBC-II. (c) IoU and Dice-Coefficient of LinkNet on AneRBC-I and AneRBC-II.

The high-performance metrics achieved by the models across both datasets emphasize their robustness and the technical soundness of the data. The UNet model’s improved performance on the AneRBC-II dataset, as compared to the AneRBC-I dataset, as shown in Tables 4 and 5, suggests that the datasets are diverse and can help to train models that generalize well. The graphical visualization regarding the segmentation validation of all datasets is shown in Fig. 10. The other graphical results related to segmentation are given in Appendix (Figs A1(a–c)).

### Classification validation

Four state-of-the-art CNN models were applied to the AneRBC datasets to validate the classification problem. We have performed classification validation on the original anemic and healthy images. We used the original image for training and testing during this classification without providing manually generated ground truth. The classification results for the AneRBC-I and AneRBC-II datasets, as obtained from the MobileNetV2, ResNet152v2 [45], VGG16, and Inceptionv3 [46] models, provide an understanding of the datasets’ utility for binary classification tasks in medical imaging. The models were executed twice, once without transfer learning to establish a baseline and then with transfer learning to check the impact of pretrained weights on performance. The detailed discussion of classification results on both datasets is given below.

#### AneRBC-I dataset classification

During classification, we explored the performance of four prominent CNNs, i.e. MobileNetV2, Resnet152V2, VGG16, and InceptionV3 on the AneRBC-I dataset. We have performed analysis on the AneRBC-I dataset both with and without the application of transfer learning. During without transfer learning experiment, all models outperformed in their training phases and acheived accuracies close to 98%. The higher training accuracies show robust feature-learning capabilities from the dataset. Among them, VGG16 outperformed with the highest validation accuracy of 94%. It also obtained a balanced precision, recall and F1 score which indicate its reliable performance across various metrics. When models were trained with transfer learning parameters, we observed a general dip in training accuracies, with Resnet152V2 achieving up to 71% accuracy from its previous high. This model also got significant increase in training and validation losses, suggesting difficulties in adapting the pretrained weights to our specific medical dataset. Meanwhile, VGG16 continued to maintain its performance with test accuracy at 86% and an F1 score of 0.92. The detail summary of all CNN model results is given in Table 6.

**Table 6.** Classification results of AneRBC-I Datasets on key metrics, including training accuracy, training loss, validation accuracy, validation loss, test accuracy, precision, recall, and F1 score for AneRBC-I dataset

	Matrices	MobileNetV2	Resnet152V2	VGG16	InceptionV3
Without transfer learning	Training accuracy	0.98	0.98	0.98	0.97
	Training loss	0.05	0.05	0.05	0.10
	Validation accuracy	0.89	0.91	0.94	0.90
	Validation loss	0.32	0.47	0.35	0.31
	Test accuracy	0.83	0.77	0.91	0.89
	Precision	0.95	0.70	0.91	0.77
	Recall	0.68	0.95	0.96	0.84
	F1 score	0.78	0.86	0.93	0.80
With transfer learning	Training accuracy	0.94	0.71	0.88	0.85
	Training loss	0.18	5.82	0.28	0.51
	Validation accuracy	0.84	0.70	0.88	0.78
	Validation loss	0.40	3.65	0.78	0.45
	Test accuracy	0.71	0.70	0.86	0.45
	Precision	0.73	0.69	0.88	0.51
	Recall	0.52	0.86	0.78	0.56
	F1 score	0.60	0.77	0.92	0.58

#### AneRBC-II dataset classification

For the AneRBC-II dataset, same models were executed with and without the application of transfer learning. All models show high training accuracies, with InceptionV3 and MobileNetV2 leading at 0.98. MobileNetV2 also showed the lowest training loss (0.04), indicating a very efficient learning process. In contrast, Resnet152V2 shows higher loss at 0.52. The validation accuracies were improved across the models, particularly for InceptionV3, which peaked at 0.97. In test scenarios, VGG16 acheived highest test accuracy at 0.94 and an F1 score of 0.95 which aligns closely with its precision and recall. Despite a lower test accuracy, Resnet152V2 managed an F1 score of 0.96, showing its ability to balance the precision and recall on AneRBC-II dataset. When applying transfer learning to the AneRBC-II dataset, all models shows decreased training accuracies and high loss rates. VGG16 still perform better and achieved test accuracy of 0.76, showing its capability to utilize features learned in different contexts. The recall rates for models like InceptionV3 and VGG16 remained high at 0.94, showing their effectiveness in identifying true positives. The detail summary of classification results on AneRBC-II dataset is given in Table 7.

**Table 7.** Classification results of AneRBC-II Datasets on key metrics, including training accuracy, training loss, validation accuracy, validation loss, test accuracy, precision, recall, and F1 score for AneRBC-II dataset

	Matrices	MobileNetV2	Resnet152V2	VGG16	InceptionV3
Without transfer learning	Training accuracy	0.98	0.97	0.97	0.98
	Training loss	0.04	0.52	0.05	0.03
	Validation accuracy	0.95	0.92	0.95	0.97
	Validation loss	0.23	0.53	0.34	0.55
	Test accuracy	0.88	0.82	0.94	0.89
	Precision	0.92	0.86	0.95	0.95
	Recall	0.93	0.93	0.95	0.98
	F1 score	0.90	0.96	0.95	0.91
With transfer learning	Training accuracy	0.89	0.73	0.86	0.73
	Training loss	0.31	8.68	0.42	0.70
	Validation accuracy	0.86	0.67	0.78	0.73
	Validation loss	0.34	4.11	0.41	0.66
	Test accuracy	0.84	0.62	0.76	0.70
	Precision	0.84	0.85	0.84	0.75
	Recall	0.86	0.91	0.94	0.94
	F1 score	0.87	0.87	0.86	0.86

**Figure 11.**
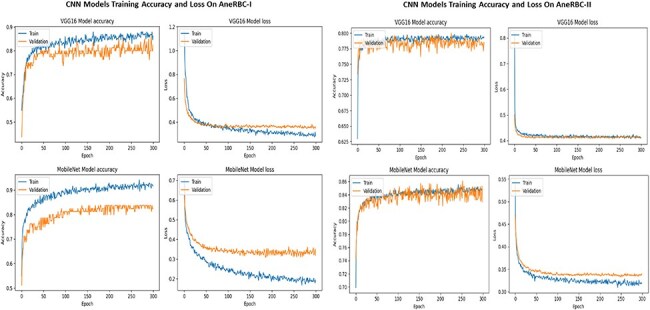
Training and validation accuracy and loss for both datasets regarding the classification of VGG16 and MobileNet.

**Figure A2.**
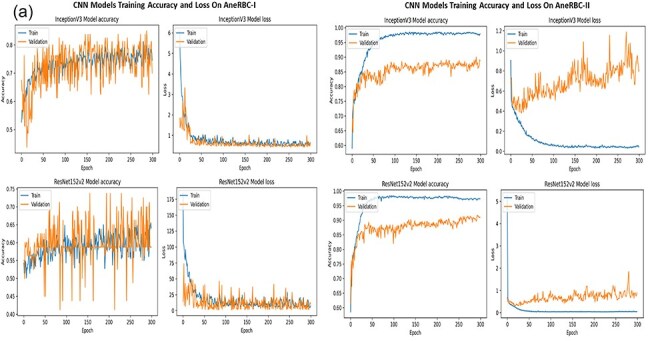

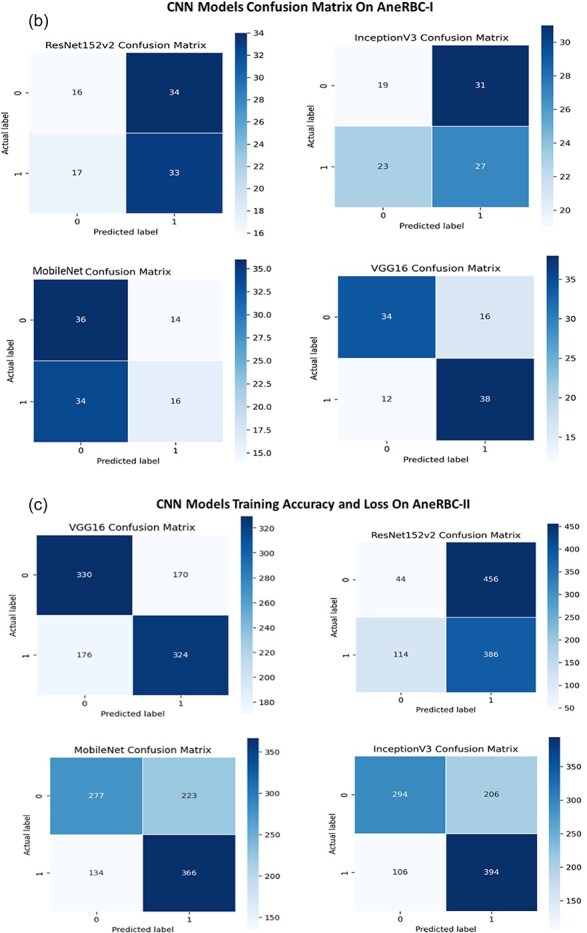
(a) Training accuracy and loss for InceptionV3 and ResNet152v2 for both datasets. (b) Confusion Matrix for InceptionV3 and ResNet152v2, VGG16, and InceptionV3 for AneRBC-I dataset. (c) Confusion Matrix for InceptionV3 and ResNet152v2, VGG16, and InceptionV3 for AneRBC-II dataset.

The consistent improvement in model performance across both datasets provides technical proof of their validity. The high precision, recall, and F1 scores achieved without transfer learning, particularly with VGG16 and InceptionV3 on the AneRBC-I dataset and InceptionV3 on the AneRBC-II dataset, demonstrate the datasets’ suitability for training classification models in the medical image domain. The fact that all models performed better without transfer learning also suggests that the datasets contain features representative of the broader medical imaging domain. The visual results for training and testing are shown in Fig. 11. Intermediate results on segmentation and classification are given in the Appendix (see Figs A1(a–c), and A2(a–c)).

## Conclusion

This study introduced the AneRBC dataset, a benchmark dataset for computer-aided anemia diagnosis using RBC images. The dataset is divided into two versions, i.e. AneRBC-I and AneRBC-II. Each contains high-resolution images with corresponding binary and RGB ground truths generated under the supervision of an expert hematologist. To validate both datasets, we have applied state-of-the-art CNN models such as UNet, LinkNet, and AttenUNet, and a cGAN architecture for segmentation. The proposed datasets were also validated for classification using MobileNetv2, ResNet152V2, VGG16, and InceptionV3. Our results demonstrate that the proposed AneRBC datasets have the potential to promote the development of automated anemia diagnostic tools by providing robust image data for training and evaluating deep learning models. Future work will focus on further annotating individual RBC classes to expand the utility of the datasets for specialized diagnostic applications.

## Data Availability

The complete image data of this dataset is available at https://data.mendeley.com/datasets/hms3sjzt7f/1 The code used for segmentation and classification is available at https://github.com/shahzadmscs/AneRBC_Segmentation_Classification_code. The code for uploading image data along with CBC and morphology reports for the training of the CNN model is available at https://github.com/shahzadmscs/AneRBC_Segmentation_Classification_code/blob/main/upload_data_for_training.ipynb
